# Endotracheal tube with tapered-type cuff for preventing ventilator-associated pneumonia: a randomized clinical trial

**DOI:** 10.1186/cc12091

**Published:** 2013-03-19

**Authors:** N Saito, T Yagi, Y Hara, H Matsumoto, K Mashiko

**Affiliations:** 1 Nippon Medical School Chiba Hokusoh Hospital, Inzai, Chiba, Japan

## Introduction

The most common cause of ventilator-associated pneumonia (VAP) is aspiration of oral secretion through the endotracheal tube (ET). Subglottic suction drainage (SSD) has been recommended as a safety measure against aspiration due to its high effectiveness. Currently, two types of cuff shape - spindle and tapered - are predominant in high-volume, low-pressure (HVLP) ETs with SSD. However, the shape most suitable for preventing dripping onto the subglottis has not been determined. The purpose of this study was to determine whether an ET with tapered-type cuff can reduce the incidence of VAP.

## Methods

After approval from the appropriate ethics committee, we conducted a single-institutional prospective randomized clinical trial on the effectiveness of using an ET with a different cuff type. Adult patients (*n = *399; ≥18 years old) were screened between April 2010 and March 2012, and 289 patients expected to require mechanical ventilation (MV) for at least 48 hours were randomized. Patients were assigned to intubation with one of the following two HVLP ETs with SSD on the experimental tube: tapered type (Taperguard™ Evac^®^; Covidien, Dublin, Ireland) or spindle type (Hi-Lo Evac^®^; Covidien). Cuff pressure measurement was carried out every 6 hours, and cuff pressure was maintained between 20 and 30 cmH_2_O. The primary outcome was VAP incidence based on semiquantitative bronchoalveolar lavage fluid culture of 3+ or phagocytosis on Gram staining in patients intubated for at least 48 hours. The other outcome was the rate of achieving an appropriate cuff pressure (20 to 30 cmH_2_O), time to VAP onset, duration of MV, duration of ICU stay, mortality, and adverse events.

## Results

The rate of microbiologically confirmed VAP was 21.7% (23/106) for the tapered-type ET patients and 21.7% (23/106) for the spindle-type ET patients (*P *= 1.00). The rate of achieving appropriate cuff pressure was 83.2% (332/1,974) for the tapered type and 82.4% (328/1,867) for the spindle type (*P *= 0.549). No significant differences between groups were observed for time to VAP onset, duration of MV, ICU stay. The incidence of reintubation due to laryngeal edema after extubation was slightly higher in patients with the tapered-type ET (11.5%, 6/52) than in patients with the spindle-type ET (2.0%, 1/49), but the difference was not significant (*P *= 0.113).

## Conclusion

Differences in cuff type and shape under identical conditions of cuff pressure control have no influence on the incidence of VAP.

